# Frequent loss of endothelin-3 (EDN3) expression due to epigenetic inactivation in human breast cancer

**DOI:** 10.1186/bcr2319

**Published:** 2009-06-15

**Authors:** Frank Wiesmann, Jürgen Veeck, Oliver Galm, Arndt Hartmann, Manel Esteller, Ruth Knüchel, Edgar Dahl

**Affiliations:** 1 Molecular Oncology Group, Institute of Pathology, University Hospital of the RWTH Aachen, Pauwelsstrasse 30, D-52074 Aachen, Germany; 2 Cancer Epigenetics Group, Cancer Epigenetics and Biology Program (PEBC), Bellvitge Institute for Biomedical Research (ICO-IDIBELL), Av. Gran Via de L'Hospitalet 199-203, E-08907 Barcelona, Spain; 3 Department of Internal Medicine IV (Haematology/Oncology), University Hospital of the RWTH Aachen, Pauwelsstrasse 30, 52074 D-Aachen, Germany; 4 Department of Pathology, University of Erlangen, Krankenhausstrasse 12, D-91054 Erlangen, Germany

## Abstract

**Introduction:**

Endothelin (EDN) signalling plays a crucial role in cell differentiation, proliferation and migration processes. There is compelling evidence that altered EDN signalling is involved in carcinogenesis by modulating cell survival and promoting invasiveness. To date, most reports have focused on the oncogenic potential of EDN1 and EDN2, both of which are overexpressed in various tumour entities. Here, we aimed at a first comprehensive analysis on EDN3 expression and its implication in human breast cancer.

**Methods:**

*EDN3 *mRNA expression was assessed by Northern blotting in normal human tissues (n = 9) as well as in matched pairs of normal and tumourous tissues from breast specimens (n = 50). *EDN3 *mRNA expression in breast cancer was further validated by real-time polymerase chain reaction (PCR) (n = 77). A tissue microarray was used to study EDN3 protein expression in breast carcinoma (n = 150) and normal breast epithelium (n = 44). *EDN3 *promoter methylation was analysed by methylation-specific PCR in breast cell lines (n = 6) before and after demethylating treatment, normal breast tissues (n = 17) and primary breast carcinomas (n = 128). EDN3 expression and methylation data were statistically correlated with clinical patient characteristics and patient outcome.

**Results:**

Loss of *EDN3 *mRNA expression in breast cancer, as initially detected by array-based expression profiling, could be confirmed by Northern blot analysis (> 2-fold loss in 96%) and real-time PCR (> 2-fold loss in 78%). Attenuated EDN3 expression in breast carcinoma was also evident at the protein level (45%) in association with adverse patient outcome in univariate (*P *= 0.022) and multivariate (hazard ratio 2.0; *P *= 0.025) analyses. Hypermethylation of the *EDN3 *promoter could be identified as the predominant mechanism leading to gene silencing. Reversion of the epigenetic lock by 5-aza-2'-deoxycytidine and trichostatin A resulted in *EDN3 *mRNA re-expression *in vitro*. Furthermore, *EDN3 *promoter hypermethylation was detected in 70% of primary breast carcinomas with significant association to loss of *EDN3 *mRNA expression (*P *= 0.005), whilst normal matched breast tissues revealed no *EDN3 *promoter methylation.

**Conclusions:**

*EDN3 *is a frequent target of epigenetic inactivation in human breast cancer, potentially contributing to imbalanced EDN signalling commonly found in this disease. The clinical implication supports the view that EDN3, in contrast to EDN1 and EDN2, may act as natural tumour suppressor in the human mammary gland.

## Introduction

Endothelins (EDNs) are widely expressed cytokines in a variety of human tissues, including brain, skeletal muscle, pancreas, small intestine, testis and colon [1]. They constitute a family of small, vasoactive, 21-amino acid peptides referred to as EDN1, EDN2 and EDN3 [2]. EDNs are synthesised as large precursor proteins that are post-translationally cleaved to the biologically active 21-amino acid form [3]. They are involved in fundamental cellular networks like cell proliferation, migration and differentiation processes [4,5] by interacting with their corresponding cell surface-bound EDN-A (EDNRA) and EDN-B (EDNRB) receptors in an autocrine and also a paracrine manner [6-8]. A balanced regulation of this EDNRA/EDNRB interplay – also referred to as the endothelin axis (ET-axis)- is essential for, for example, homing processes to tissue destinations, where cells differentiate into numerous lineages such as the peripheral nervous system, structural and connective tissue components, cardiac cells or pigment-producing melanocytes [9].

There is now compelling evidence that imbalanced regulation of the ET-axis is implicated in human carcinogenesis, tumour progression and neo-angiogenesis [8,10-12]. During malignant cell transformation, the basic tissue architecture, which is maintained by basement membrane delineation, becomes disrupted [8]. This indicates the presence of crucial mediators that trigger the exchange of growth factors between the participating cells at the tumour invasion field. Essentially, such growth factor release is thought to enhance invasiveness, stimulate cell migration and promote neo-vascularisation [8]. Multiple signal transduction pathways are affected downstream from EDNRA/B. In the case of interaction of EDNs with EDNRA, a pertussis toxin-insensitive G protein becomes activated and promotes stimulation of phospholipase C, resulting in the transactivation of the mitogen-activated protein kinase pathway [13]. Second, EDN1 and EDN2 binding to EDNRA can activate p125 focal adhesion kinase and paxillin, both of which have been associated with increased tumour cell invasion. Moreover, EDNs are able to transduce the activation of anti-apoptotic signals through phosphatidylinositol-3-kinase and to stimulate neo-angiogenesis through vascular endothelial growth factor signalling [14]. These multiple ET-axis pathway implications may explain its various impairments of normal cellular integrity in case of an aberrant shift from balanced to imbalanced EDN signalling.

Previously, EDN1 and EDN2 were found to be commonly overexpressed in a broad range of human tumour entities [8,11,12]. So far, most reports have focused on the role of EDN1 binding to EDNRA and its effects on tumour growth and neo-angiogenesis [8,11,13,15]. A role similar to that of EDN1 has been described for EDN2 in human breast cancer. Increased expression of EDN1 and EDN2, but not of EDN3, induced chemotaxis of breast cancer cells and increased tumour cell invasion through the basement membrane [4], although conflicting results have been reported by others [16]. In line with this, previous reports described a compensatory effect of EDN3 by negatively modulating the effects transduced by EDN1 [17] and demonstrated that downregulation of EDN3 is associated with upregulation of EDN1 in human tissues [18].

However, a comprehensive analysis of EDN3 expression in normal and cancerous breast tissues and its potential implication in human breast cancer has not been published so far. In our study, we investigated for the first time *EDN3 *mRNA and protein expression in a large number of primary breast tissues and breast cell lines. Furthermore, we identified the molecular mechanism by which EDN3 expression is deregulated in breast carcinomas.

## Materials and methods

### Cryo-conserved clinical patient material

Cryo-conserved clinical samples were obtained from breast cancer patients treated by primary surgery at the University Hospitals of Aachen, Düsseldorf and Regensburg. Patients receiving neo-adjuvant chemotherapy and patients with recurrent breast cancer were excluded. Resected tissue was snap-frozen in liquid nitrogen immediately after surgery. Only samples containing more than 70% of tumourous cells in haematoxylin/eosin-stained control sections were further processed (n = 128). For 17 samples, macroscopically normal breast tissues containing at least 30% of epithelial cells were available. In all cases, two board-certified pathologists agreed on the diagnosis of breast cancer. Tumour histology was determined according to the criteria of the World Health Organization (2003), whereas disease stage was assessed according to the UICC (Union Internationale contre le Cancer) [19]. Tumours were graded according to Bloom and Richardson, as modified by Elston and Ellis [20]. All patients gave informed consent for retention and analysis of their tissue for research purposes, and the institutional review boards of the participating centres approved the study. For 98 patients, follow-up data were available with a median time of 63 months (range 1 to 124 months). Patient characteristics of this cohort are summarised in Table 1.

**Table 1:** Clinicopathological parameters of cryo-conserved breast cancer specimens (n = 128)

Variables	Number^a^	Percentage
Clinicopathological factors		
Age at diagnosis^b^		
< 58 years	64	50.0
≥ 58 years	64	50.0
Tumour size^c^		
pT1	44	34.4
pT2	55	43.0
pT3	6	4.7
pT4	11	8.6
pTx	12	9.4
Lymph node status^c^		
pN0	57	44.5
pN1–3	51	39.8
pNx	20	15.6
Histological grade		
G1	10	7.8
G2	58	45.3
G3	49	38.3
NA	11	8.6
Histological type		
Ductal	103	80.5
Lobular	15	11.7
Other	6	4.7
NA	4	3.1
Immunohistochemistry		
Oestrogen receptor		
Negative (IRS^d ^≤ 2)	33	25.8
Positive (IRS > 2)	85	66.4
NA	10	7.8
Progesterone receptor		
Negative (IRS^d ^≤ 2)	33	25.8
Positive (IRS > 2)	85	66.4
NA	10	7.8

Due to rounding, percentages may not sum to 100. ^a^Only female patients with primary invasive breast cancer were included. ^b^Median 58 years, range 28 to 87 years. ^c^According to the UICC (Union Internationale contre le Cancer): *TNM Classification of Malignant Tumours *[19].^d^Immunoreactivity score (IRS) according to Remmele and Stegner [24]. NA, information not available.

### Formalin-fixed paraffin-embedded clinical patient material

A tissue microarray (TMA) was created as described previously by Bubendorf and colleagues [21]. The formalin-fixed paraffin-embedded (FFPE) tissue sections were obtained from the archive of the Institute of Pathology, University of Regensburg, Germany. In all cases, two board-certified pathologists agreed on the diagnosis of breast cancer. Patients receiving neo-adjuvant chemotherapy and patients with recurrent breast cancer were excluded. All patients gave informed consent for retention and analysis of their tissues for research purposes, and the institutional review board of the participating centre approved the study. The TMA consisted of 150 primary tumours from malignant breast tissue and 44 normal breast specimens. Follow-up data were available for 146 patients with a median time of 77 months (range 1 to 148 months). Detailed tumour characteristics of this cohort are listed in Table 2.

**Table 2:** Clinicopathological parameters of formalin-fixed paraffin-embedded breast cancer specimens (n = 150)

Variables	Number^a^	Percentage
Clinicopathological factors		
Age at diagnosis^b^		
≤ 59 years	83	55.3
> 59 years	67	44.7
Tumour size^c^		
pT1	48	32.0
pT2	71	47.3
pT3	9	6.0
pT4	20	13.3
pTx	2	1.3
Lymph node status^c^		
pN0	67	44.7
pN1	35	23.3
pN2	25	16.7
pN3	20	13.3
pNx	3	2.0
Histological grade		
G1	13	8.7
G2	70	46.7
G3	66	44.0
NA	1	0.7
Histological type		
Ductal	122	81.3
Lobular	12	18.0
Other	16	10.7
Tumour focality		
Unifocal	130	86.7
Multifocal	19	12.7
NA	1	0.7

Immunohistochemistry		
Oestrogen receptor		
Negative (IRS^d ^≤ 2)	38	25.3
Positive (IRS > 2)	83	55.3
NA	29	19.3
Progesterone receptor		
Negative (IRS^d ^≤ 2)	85	56.7
Positive (IRS > 2)	40	26.7
NA	25	16.7
Her2 status		
Negative (DAKO score 0; 1+)	105	70.0
Positive (DAKO score 2+; 3+)	23	15.3
NA	22	14.7

Due to rounding, percentages may not sum to 100. ^a^Only female patients with primary invasive breast cancer were included. ^b^Median 59 years, range 29 to 82 years. ^c^According to the UICC (Union Internationale contre le Cancer): *TNM Classification of Malignant Tumours *[19]. ^d^Immunoreactivity score (IRS) according to Remmele and Stegner [24]. NA, information not available.

### Breast cell lines

The non-cancerous breast cell lines MCF10A and MCF12A as well as the cancerous breast cell lines MCF7, SKBR3, MDA-MB231 and BT20 were obtained from the American Type Culture Collection (Manassas, VA, USA) and cultured as recommended by the vendor.

### Northern blot expression analysis

Expression of *EDN3 *mRNA in various human tissues was tested using the commercial multiple-tissue Northern (MTN) blots I and II (Clontech, Heidelberg, Germany), containing 2 μg of poly A^+ ^RNA per lane from 16 different human tissues (that is, blot I: heart, whole brain, placenta, lung, liver, skeletal muscle, kidney and pancreas; blot II: spleen, thymus, prostate, testis, ovary, small intestine, colon [no mucosa] and peripheral blood lymphocytes). Hybridisation was performed using 25 ng of an *EDN3*-specific 722-base pair (bp) polymerase chain reaction (PCR) product derived from (GenBank accession number NM_000114.2) (position: 968 to 1,707), which was verified by sequence analysis. ^32^P-labelling of the DNA probe was achieved using the Megaprime DNA Labeling System (Amersham Biosciences, now part of GE Healthcare, Little Chalfont, Buckinghamshire, UK), and hybridisation was performed in accordance with the recommendation the manufacturer. The cancer profiling array (CPA) I (Clontech) is a matched tumour/normal expression array consisting of cDNA synthesised from 50 breast carcinomas, 50 normal breast tissues and 3 breast cancer lymph node metastasis specimens. Hybridisation was performed in accordance with the recommendations the manufacturer as described above for the MTN blots. Hybridisation signals of both, MTN blots and the CPA, were evaluated by use of a STORM-860 phosphoimager (Molecular Dynamics, now part of GE Healthcare). Intensity ratios were calculated after normalising signals against the background.

### Nucleic acid extraction

Frozen tissue samples and cell line pellets were dissolved in lysis buffer for subsequent DNA isolation using the QIAmp DNA Mini kit (Qiagen, Hilden, Germany) or for total RNA isolation by using TRIzol reagent (Invitrogen Corporation, Carlsbad, CA, USA) in accordance with the protocol supplied by the manufacturers.

### Reverse transcription of RNA

Of the extracted total RNA, 1 μg was reverse-transcribed using the Reverse Transcription System (Promega Corporation, Madison, WI, USA) by applying a mix of oligo-dT and pdN_(6)_-hexamer primers (1:2). The obtained cDNA was diluted (20 ng/μL) and test-amplified using intron-spanning primers for glyceraldehyde-3-phosphate-dehydrogenase (*GAPDH*). Primer sequences are provided in Table 3. PCRs were initiated as 'Hot Start' PCR at 95°C for 5 minutes and a hold at 80°C before the addition of 1 unit of Go*Taq *DNA polymerase (Promega Corporation). Cycle conditions were 95°C for 5 minutes, 35 cycles of 95°C for 1 minute, 60°C for 1 minute, 72°C for 1 minute and a final extension at 72°C for 10 minutes. PCR analyses were carried out in a PTC-200 cycler (Bio-Rad Laboratories, Inc., formerly MJ Research, Hercules, CA, USA). Amplificates were evaluated under ultraviolet light after 2% agarose gel electrophoresis containing ethidium bromide. Only samples yielding a specific 510-bp amplificate were further subjected to real-time PCR.

**Table 3:** Oligonucleotide primers used in this study

	Sequence (5'↔3')	TA, °C	Cycles	Product, base pairs
RT-PCR				
GAPDH				
	Forward: TGGTCACCAGGGCTGCTT	60	35	510
	Reverse: GTCTTCTGGGTGGCAGTGAT			

Real-time PCR				
GAPDH				
	Forward: GAAGGTGAAGGTCGGAGTCA	58	40	108
	Reverse: TGGACTCCACGACGTACTCA			
EDN1				
	Forward: GCTCGTCCCTGATGGATAAA	58	40	216
	Reverse: TTCCTGCTTGGCAAAAATTC			
EDN2				
	Forward: TTGGACATCATCTGGGTGAA	58	40	229
	Reverse: CTGTAGTGGCCCCTGTCTTG			
EDN3				
	Forward: ATTGCCACCTGGACATCATT	58	40	179
	Reverse: GCAGGCCTTGTCATATCTCC			

Methylation-specific PCR				
EDN3-U				
	Forward: TTTGGGAGGTGATTTTTAGTGTGTTT	60	35	144
	Reverse: ACCCATCCCTACACAAAACTAACCA			
EDN3-M				
	Forward: TGGGAGGCGATTTTTAGTGCGTTC	60	35	140
	Reverse: CCATCCCTACGCGAAACTAACCG			

EDN, endothelin; EDN3-M, endothelin-3 methylated; EDN3-U, endothelin-3 unmethylated; GAPDH, glyceraldehyde-3-phosphate-dehydrogenase; PCR, polymerase chain reaction; RT-PCR, reverse transcription-polymerase chain reaction; TA, annealing temperature.

### Semi-quantitative real-time polymerase chain reaction

The Roche LightCycler system was used for semi-quantitative light cycler analysis in combination with the LightCycler DNA Master SYBR Green I Kit (Roche, Mannheim, Germany) as previously described [22]. Gene expression was quantified by the comparative cycle threshold (C_T_) method, normalising C_T _values to the housekeeping gene *GAPDH *and calculating relative expression values [23]. A commercially available normal breast cDNA pool (Clontech) was used as a breast reference standard. Primer sequences are listed in Table 3.

### Immunohistochemistry

Paraffin sections of 2 μm were deparaffinised in xylene followed by rehydration in a decreasing ethanol series. Antigen retrieval was performed by pre-treatment in boiling citrate buffer (pH 6.0) in a microwave oven for 30 minutes (200 W). Immunohistochemistry (IHC) was performed using an NEXES Immuno Stainer (Ventana Medical Systems, Inc., Tucson, AZ, USA) in accordance with the specifications of the manufacturer. A goat polyclonal EDN3-specific antibody (sc-21628; Santa Cruz Biotechnology, Inc., Santa Cruz, CA, USA) was used in a 1:150 dilution by using the ChemMate Envision Kit (DAKO, Hamburg, Germany). Counterstaining was performed by using Mayer's haematoxylin. The incubation with primary antibodies was omitted in negative controls. All analysed samples were stained without the knowledge of histopathological data. Cytoplasmic protein staining was semi-quantitatively scored by an experienced breast pathologist according to the well-established scoring system developed by Remmele and Stegner [24]. To verify staining specificity, the primary antibody was incubated with a 200 molar excess of blocking peptide (sc-21628 P; Santa Cruz Biotechnology, Inc.) for 2 hours prior to its application on test samples.

### CpG island prediction

*EDN1*, *EDN2 *and *EDN3 *genomic nucleotide sequences were taken from the Ensembl database and analysed for promoter CpG (cytosine-phosphate-guanine dinucleotide) islands in accordance with the method of Li and Dahiya [25]. A fragment of 2 kb in size, beginning 1 kb 5'-upstream from the annotated transcription start site (TSS) and ending 1 kb 3'-downstream, was analysed by applying the following criteria: island size of greater than 200 bp, guanine/cytosine content of greater than 60% and observed/expected CpG ratio of greater than 0.6.

### Bisulphite modification and methylation-specific polymerase chain reaction

Of the genomic DNA, 1 μg was bisulphite-modified using the EZ DNA Methylation Kit (Zymo Research Corporation, Orange, CA, USA) and eluted in 20 μL of Tris buffer (10 mM). Methylation-specific PCR (MSP) was performed in accordance with the method of Herman and colleagues [26]. One microlitre of bisulphite-treated DNA was amplified using MSP primers that specifically recognise either the unmethylated or methylated *EDN3 *promoter sequence after bisulphite conversion (Table 3). To achieve high accuracy, each primer was designed to cover three CpG sites of template DNA. Commercially available universal poly-methylated DNA and unmethylated DNA (EpiTect Control DNA; Qiagen) were used as positive controls for methylated and unmethylated *EDN3 *sequences, respectively. MSP products were visualised under ultraviolet light after 3% low-range ultra agarose gel electrophoresis containing ethidium bromide (Bio-Rad Laboratories, Inc.). Promoter methylation status was interpreted in a binary qualitative fashion.

### *In vitro *demethylating treatment

Cells were seeded at a density of 3 × 10^4 ^cells/cm^2 ^in a six-well plate. The demethylation agent 5-aza-2'-deoxycytidine (DAC) (Sigma-Aldrich, Deisenheim, Germany) was added to a final concentration of 1 μM in fresh medium at days 1, 2 and 3 after seeding. Additionally, cells were exposed to 300 nM trichostatin A (TSA) (Sigma-Aldrich) on day 3 for 24 hours. Control cells without DAC/TSA were supplied with fresh medium on days 1, 2 and 3. DNA and RNA were extracted on day 4 as mentioned above.

### Statistical evaluations

SPSS version 14.0 (SPSS Inc., Chicago, IL, USA) was used for statistical analyses. All tests were performed two-tailed, and *P *values of below 0.05 were considered statistically significant. The non-parametric Mann-Whitney *U *test and the Student *t *test (paired and unpaired) were used to compare expression results between cancer tissues and normal tissues or between *EDN3 *mRNA expression and *EDN3 *methylation status. Contingency table analysis and Fisher exact tests were used to study the statistical association between clinicopathological factors and EDN3 protein expression or *EDN3 *promoter methylation status. Survival curves comparing patients with or without any of the factors were calculated using the Kaplan-Meier method, with significance evaluated by log-rank statistics. Breast cancer-specific survival (CSS) was measured from the day of surgery until tumour-related death and was censored for patients alive at last contact or in case of death unrelated to the tumour. Disease-free survival (DFS) was measured from surgery until disease relapse and censored for patients alive without evidence of relapse at the last follow-up. For EDN3 protein expression, a multivariate Cox proportional hazard model was employed to assess the relative risks on patient CSS and to test for independent prognostic relevance of clinical/investigational factors. Only patients for whom the status of all selected variables was known were included in the proportional hazard model (n = 121). The limit for reverse-selection procedures was *P *= 0.2. The proportionality assumption for all variables was assessed with log-negative-log survival distribution functions. For analyses, the following variables were categorised into binary values: small-sized (pT1) versus large-sized (pT2 to pT4), node-negative (pN0) versus node-positive (pN1 to pN3) and low-grade (G1 and G2) versus high-grade (G3) tumours.

## Results

### *EDN3 *mRNA is differentially expressed in human breast cancer

As previously reported, we performed *in silico *Northern blot analysis and RNA array-based expression profiling to identify novel candidate genes differentially expressed in human breast cancer [27,28]. In the latter approach, *EDN3 *was detected as one of the most frequently downregulated genes, showing substantial expression loss in 63% of breast carcinomas (data not shown). Therefore, we started a detailed analysis on EDN3 expression and its potential implication in human breast cancer.

**Figure 1:**
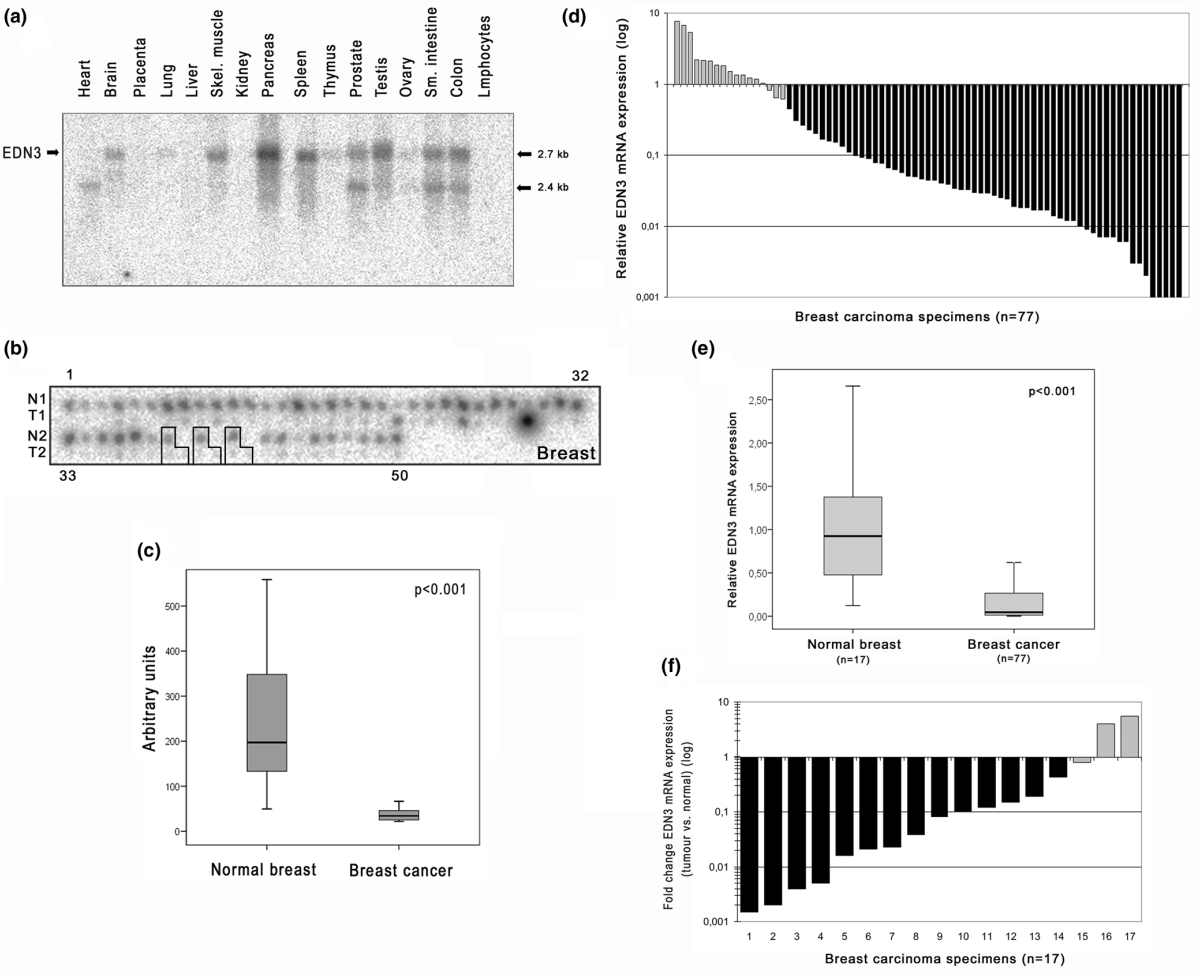
Differential expression of *EDN3 *mRNA in human breast cancer. **(a) **A multiple tissue Northern blot hybridised with an *EDN3*-specific probe indicates ubiquitous expression of *EDN3 *mRNA in a variety of normal tissue types. In some tissues, both major *EDN3 *transcript variants are expressed (2.4 and 2.7 kb). **(b) **The same probe was hybridised to a breast cancer array containing cDNA pairs from 50 breast carcinoma tissues (T), 50 matched normal breast tissues (N) and 3 metastatic tissues. The outlined groups represent matched pairs, including the metastatic deposit, and consecutive numbers indicate specimen spots on the array. **(c) **Box plot demonstrating significant downregulation of *EDN3 *expression between normal and tumourous breast tissues (*P *< 0.001, Student paired *t *test; extreme value of specimen #29 omitted). Horizontal lines indicate group medians, and boxes indicate 25% to 75% quartiles, range, peak and minimum. **(d) **Real-time polymerase chain reaction analysis demonstrated loss of *EDN3 *expression in 78% of breast carcinomas by FC2 (fold change of greater than 2) (black bars) whilst 22% showed no deregulation (grey bars). **(e) **Box plot demonstrating the different distributions of *EDN3 *expression among normal breast tissues and breast carcinomas (*P *< 0.001, Mann-Whitney *U *test). Horizontal lines indicate group medians, and boxes indicate 25% to 75% quartiles, range, peak and minimum. **(f) **Fold changes of *EDN3 *expression in 17 matched pairs of normal breast and breast carcinoma samples revealed loss of expression in 13 cases (76%) and a median expression change of 13-fold. EDN3, endothelin-3.

An initial Northern blot analysis of normal human tissues demonstrates that *EDN3 *mRNA is abundantly expressed in a variety of non-malignant tissues, including pancreas, spleen, prostate, testis, small intestine and colon, with two major mRNA transcripts of 2.4 and 2.7 kb in size (Figure 1a). A breast cancer dot blot cDNA array was then hybridised with the same *EDN3*-specific probe. This showed a clear loss of *EDN3 *mRNA expression, as defined by a fold change (tumour versus normal) of greater than 2 (FC2), in 96% (48 of 50) of the analysed breast carcinoma samples and also in all three corresponding lymph node metastases (*P *< 0.001) (Figure 1b, c). To confirm this result, *EDN3 *mRNA expression was also assessed by real-time PCR in a set of 77 breast tumour tissues and 17 corresponding normal breast tissues. In breast carcinomas, downregulation of *EDN3 *expression by FC2 could be detected in 60 of 77 cases (78%; median: 21-fold) as compared with the normal breast reference standard (Figure 1d). This downregulation was still evident when comparing *EDN3 *expression among all tumour and normal breast tissues as illustrated by box plot analysis (*P *< 0.001) (Figure 1e) as well as when comparing fold changes of *EDN3 *expression among the 17 matched pairs (Figure 1f). In the latter analysis, 14 of 17 pairs (82%) showed *EDN3 *downregulation by FC2, with a median expression change of 13-fold.

### Differential EDN3 protein expression in human breast cancer

**Figure 2:**
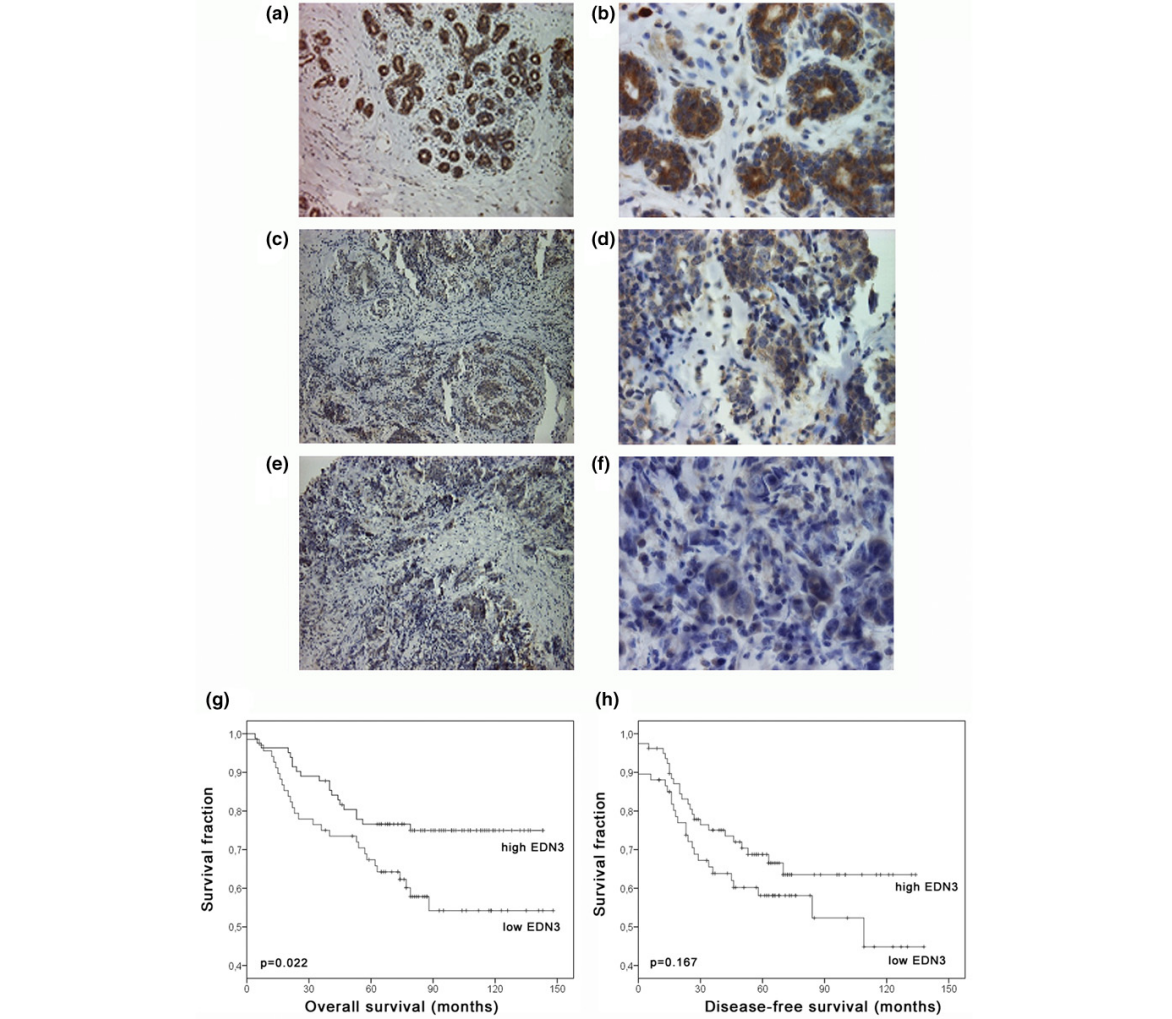
Differential EDN3 protein expression in human breast cancer and its clinical relevance. **(a) **Abundant EDN3 protein expression in normal breast epithelium. **(b) **Magnification of specimen shown in (a). **(c) **Representative invasive-ductal carcinoma showing moderate EDN3 protein expression. **(d) **Magnification of specimen shown in (c). **(e) **Representative invasive-ductal carcinoma with substantial loss of EDN3 protein expression. **(f) **Magnification of specimen shown in (e). Kaplan-Meier curves indicating that retaining high EDN3 protein expression (immunoreactivity score of at least 8) in the tumour is significantly associated with more favourable **(g) **breast cancer-specific survival (*P *= 0.022, log-rank test) but not **(h) **disease-free survival. Magnifications: ×100 (a, c, e), ×200 (b, d, f). EDN3, endothelin-3.

To analyse whether loss of EDN3 expression in breast cancer is also evident on the protein level, we used a TMA comprising 150 invasive breast carcinomas and 44 normal breast tissue specimens. The specificity of the antibody applied was determined by the simultaneous use of blocking peptide against EDN3 in normal breast samples, which showed a clear decrease of overall EDN3 staining (Additional data file 1). EDN3 protein was clearly detectable in 75.0% (32 of 44) of normal breast tissue samples analysed (Figure 2a, b), as defined by an immunoreactivity score (IRS) of at least 8. The mean normal expression was determined to be IRS = 8.2 (± 3.8 standard deviations [SDs]). Expression was predominantly localised in luminal and basal epithelial cells of the normal breast and was weakly detectable in normal stromal compartments. In contrast, invasive breast carcinomas showed complete loss or reduced EDN3 expression (IRS < 8) in 45.3% (68 of 150) of cases (Figure 2c, d) and abundant EDN3 expression in 54.7% (82 of 159) (Figure 2e, f). Mean EDN3 expression in invasive breast carcinomas was determined to be IRS = 6.7 (± 4.0 SDs). EDN3 protein was rarely observed in tumour stroma (that is, was detectable only in those stromal cells adjacent to tumour cells with strong EDN3 expression). The difference of EDN3 expression between tumours and normal breast tissues was statistically significant (Mann-Whitney *U *test: *P *= 0.037; Student unpaired *t *test: *P *= 0.039).

### Loss of EDN3 expression is associated with adverse patient outcome in human breast cancer

To investigate a potential clinical relevance of EDN3 in breast cancer, we first analysed whether EDN3 protein expression is associated with clinicopathological parameters. In a bivariate analysis, differential EDN3 expression was not associated with patient age at diagnosis, tumour size, lymph node metastasis, histological grade, histological type, tumour focality or oestrogen or progesterone receptor status (Table 4). Interestingly, in a univariate survival analysis, we found a significant association between low EDN3 expression and unfavourable CSS (*P *= 0.022) (Figure 2g and Table 5). Patients with low EDN3 expression in the tumour had a mean CSS of 99 months (95% confidence interval [CI] 85 to 113 months) as compared with patients with tumours showing high EDN3 expression, which had a prolonged mean CSS of 116 months (95% CI 106 to 126 months). The visual impression of the Kaplan-Meier curves suggests also an association of EDN3 expression with DFS (Figure 2h), but this was statistically not significant (*P *= 0.167) (Table 5). Next, we performed a multivariate Cox regression analysis to test for independent significance of EDN3 expression as a prognostic factor in patient CSS (Table 6). Patient age at diagnosis, tumour size, lymph node metastasis, histological grade and EDN3 expression were included in the model. After reverse selection, patient age, lymph node status, grade and EDN3 expression remained significant in the Cox model, with low EDN3 expression displaying a twofold elevated risk of dying from breast cancer (hazard ratio 1.98, 95% CI 1.09 to 3.61; *P *= 0.025).

**Table 4:** Clinicopathological factors in relation to EDN3 protein expression

	EDN3 expression			
Variables	Number^a^	Low (IRS < 8)	High (IRS ≥ 8)	*P *value^b^

Clinicopathological factors				
Age at diagnosis				
≤ 59 years	83	36	47	0.632
> 59 years	67	32	35	
Tumour size^c^				
pT1	48	21	27	0.728
pT2 to pT4	100	47	53	
Lymph node status^c^				
pN0	67	29	38	0.742
pN1 to pN3	80	37	43	
Histological grade				
G1 and G2	83	35	48	0.408
G3	66	33	33	
Histological type				
Ductal	122	53	69	0.385
Lobular	12	5	7	
Other	16	10	6	
Tumour focality				
Unifocal	130	61	69	0.467
Multifocal	19	7	12	
Oestrogen receptor				
Negative (IRS^d ^≤ 2)	38	19	19	0.698
Positive (IRS > 2)	83	45	38	
Progesterone receptor				
Negative (IRS^d ^≤ 2)	85	41	44	0.704
Positive (IRS > 2)	40	21	19	
Her2 status				
Negative (IHC: 0; 1+)	105	46	59	0.169
Positive (IHC: 2+; 3+)	23	14	9	

^a^Only female patients with primary invasive breast cancer were included. ^b^Fisher exact test (two-sided). ^c^According to the UICC (Union Internationale contre le Cancer): *TNM Classification of Malignant Tumours *[19].^d^Immunoreactivity score (IRS) according to Remmele and Stegner [24]. EDN3, endothelin-3; IHC, immunohistochemistry.

**Table 5:** Clinicopathological factors in relation to disease-free and breast cancer-specific survival

	Disease-free survival			Breast cancer-specific survival		
	Number^a^	Events	*P *value^b^	Number^a^	Events	*P *value^b^

Variables						
Clinicopathological factors						
Age at diagnosis						
≤ 59 years	83	28	0.163	83	19	0.005^c^
> 59 years	63	25		67	29	
Tumour size^d^						
pT1	47	11	0.007^c^	48	9	0.013^c^
pT2 to pT4	97	42		100	39	
Lymph node status^d^						
pN0	67	9	< 0.001^c^	67	10	< 0.001^c^
pN1 to pN3	76	41		80	35	
Histological grade						
G1 and G2	81	23	0.008^c^	83	19	0.002^c^
G3	64	30		66	29	
Histological type						
Ductal	120	46	0.376	122	40	0.474
Lobular	11	4		12	5	
Other	15	3		16	3	
Tumour focality						
Unifocal	127	44	0.158	130	39	0.082
Multifocal	18	9		19	9	
Oestrogen receptor						
Negative (IRS^e ^≤ 2)	38	16	0.351	38	16	0.101
Positive (IRS > 2)	79	25		83	22	
Progesterone receptor						
Negative (IRS^e ^≤ 2)	81	32	0.214	85	32	0.056
Positive (IRS > 2)	40	11		40	8	
Her2 status						
Negative (IHC: 0; 1+)	101	38	0.840	105	33	0.445
Positive (IHC: 2+; 3+)	23	9		23	9	
EDN3 expression						
Low (IRS^e ^< 8)	67	28	0.167	68	28	0.022^c^
High (IRS ≥ 8)	79	25		82	20	

^a^Only female patients with primary invasive breast cancer were included. ^b^Log-rank test. ^c^Significant data. ^d^According to the UICC (Union Internationale contre le Cancer): *TNM Classification of Malignant Tumours *[19].^e^Immunoreactivity score (IRS) according to Remmele and Stegner [24]. EDN3, endothelin-3; IHC, immunohistochemistry.

**Table 6:** Multivariate Cox regression analysis of clinicopathological factors potentially influencing breast cancer-specific survival, including EDN3 protein expression

Variable	Value	*P *value	Hazard ratio (HR)	95% confidence interval of HR	
				Lower	Upper

Age					
Continuous		0.005^a^	1.04^a^	1.01^a^	1.06^a^
pT					
pT1	0		1.00		
pT2 to pT4	1	0.109	1.87	0.87	4.04
pN					
pN0	0		1.00		
pN1 to pN3	1	0.002^a^	3.12^a^	1.53^a^	6.33^a^
Grade					
G1 and G2	0		1.00		
G3	1	0.013^a^	2.17^a^	1.18^a^	3.99^a^
EDN3 expression					
High	0		1.00		
Low	1	0.025^a^	1.98^a^	1.09^a^	3.61^a^

The variables were categorised according to Tables 4 and 5, except for age (continuous). ^a^Significant data. EDN3, endothelin-3.

### Loss of *EDN3 *mRNA expression in breast cancer cell lines

**Figure 3:**
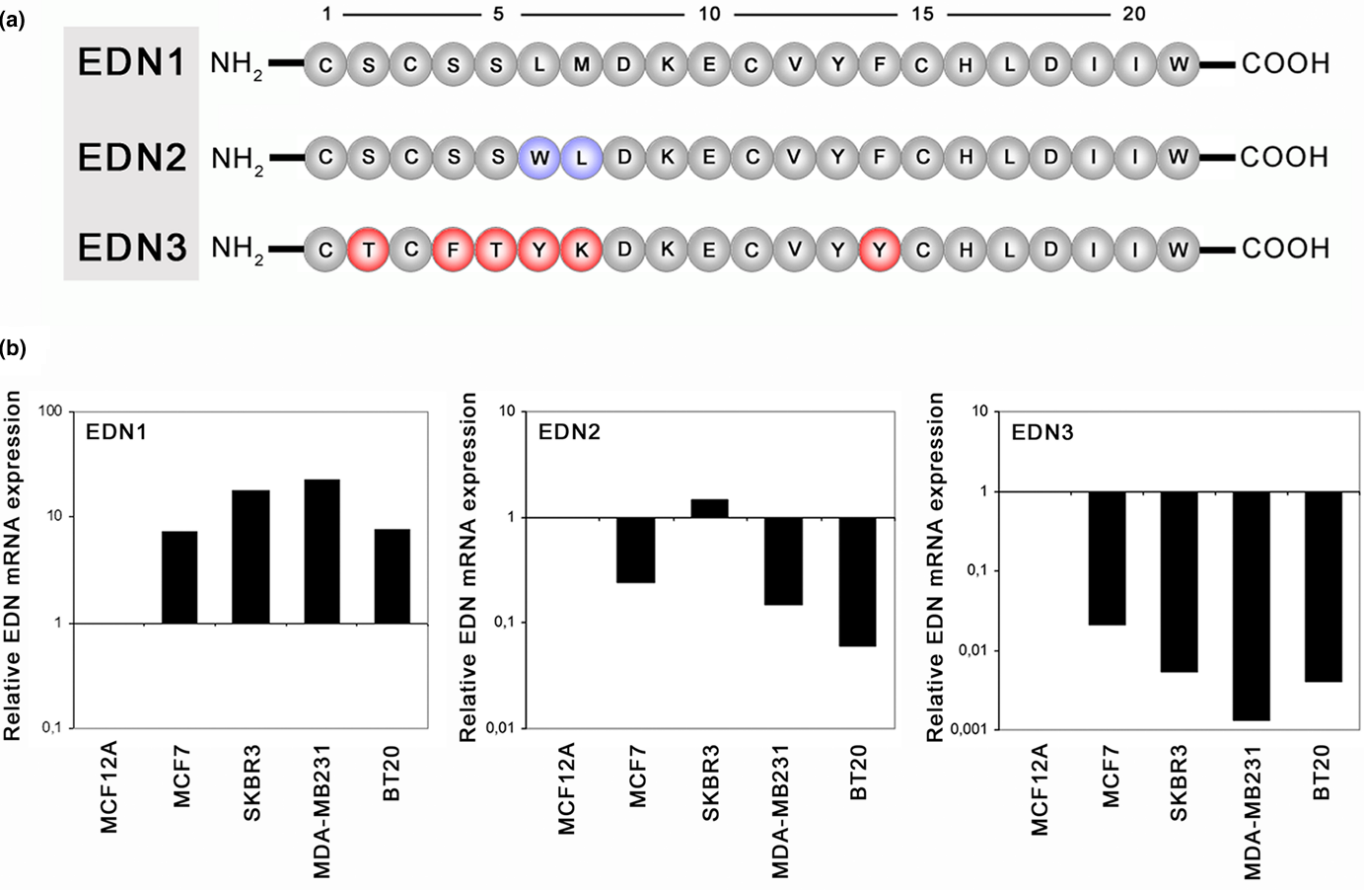
Endothelin (EDN) mRNA expression analysis in breast cell lines. **(a) **Alignment of the primary amino acid (aa) structure of human EDN1, EDN2 and EDN3 after post-translational cleavage to the biologically active 21-aa form [4]. The secondary structure consists of single α-helices containing two disulphide bonds that hold them in a conical spiral shape, joining cysteins at positions 1–15 and 3–11 [53]. EDN3 structure differs mainly in the NH_2_-terminal region from the structure of EDN1 and EDN2. This region forms a bulge out of the basic EDN structure and has been reported to represent a major domain for binding specificity to EDN receptors and thus is critical in endothelin signalling activity [29]. Numbers indicate consecutive aa residues, and aa residues are indicated in universal single-letter aa code. **(b) **Real-time polymerase chain reaction comparing *EDN3 *expression with *EDN1 *and *EDN2 *expression. While *EDN1 *is overexpressed in cancerous breast cell lines (MCF7, SKBR3, MDA-MB231 and BT20), *EDN3 *expression is abrogated in the same malignant cells as compared with MCF12A cells (set to 1 in each diagram).

The NH_2_-terminal peptide structure of EDN3 differs considerably in essential amino acids between residues 2 and residues 4 to 7 from those of EDN1 and EDN2 (Figure 3a). This region bulges out of the basic EDN structure and has been reported to represent a major domain for binding specificity to EDN receptors and thus is critical in EDN signalling activity [29]. Because we found that *EDN3 *expression is frequently lost in breast cancer tissues, we next compared *EDN1*, *EDN2 *and *EDN3 *mRNA expression in breast cell lines in parallel. *EDN1 *was much more strongly expressed in all cancerous cell lines (MCF7, SKBR3, MDA-MB231 and BT20) than in non-cancerous MCF12A cells (Figure 3b), showing a mean upregulation of 13.9-fold (± 7.8 SDs). *EDN2 *was more strongly expressed in SKBR3 cells but less expressed in MCF7, MDA-MB231 and BT20 as compared with MCF12A cells. *EDN3 *expression, however, was clearly downregulated in all cancerous cell lines as compared with non-cancerous cells, revealing a mean expression of 0.008 (± 0.009 SDs) of that found in MCF12A cells (set to 1). Therefore, upregulation of *EDN1 *appears to coincide with downregulation of *EDN3 *in breast cancer cell lines.

### Methylation of the *EDN3 *promoter in breast cancer cell lines

**Figure 4:**
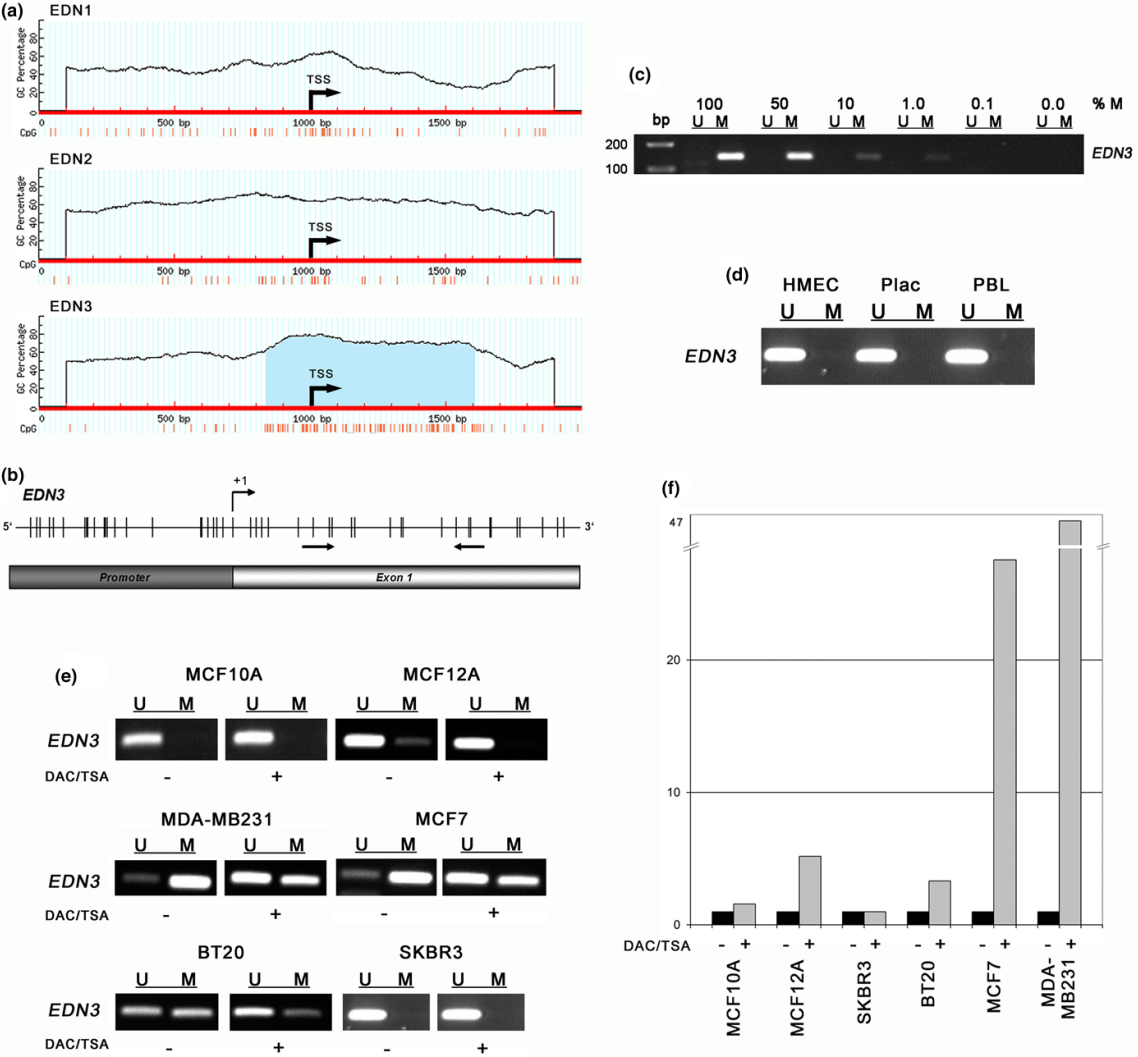
*EDN3 *promoter methylation analysis in breast cell lines. **(a) **Prediction of CpG islands in EDN family genes. A 2-kb genomic nucleotide sequence of *EDN1*, *EDN2 *and *EDN3 *was analysed with MethPrimer software [25]. A region of particularly high CpG density (red vertical bars) in the *EDN3 *nucleotide sequence proximal to the transcription start site (TSS) was identified as a CpG island (blue shaded). **(b) **Schematic representation of the *EDN3 *gene fragment that has been analysed for methylation. Arrows indicate hybridisation sites of methylation-specific polymerase chain reaction (MSP) primers, +1 indicates TSS, and vertical bars depict CpG dicnucleotides. **(c) **A dilution series of *in vitro *poly-methylated DNA with unmethylated DNA demonstrates the sensitivity of the applied MSP primers, which detect at least 1% of methylated DNA (M) in a background of unmethylated DNA in MSPs. **(d) **The *EDN3 *promoter is unmethylated in HMEC and non-malignant tissues. **(e) ***EDN3 *methylation analysis in breast cancer cell lines before (-) and after (+) treatment with demethylating (DAC) and histone reacetylating (TSA) drugs. In cell lines originally showing methylated *EDN3 *promoter alleles (MCF12A, MDA-MB231, MCF7 and BT20), a conversion of methylation was achieved as indicated by a gain of signal strength for non-methylation (U) and loss of signal strength for methylation (M), whereas in originally unmethylated cells (MCF10A and SKBR3), the treatment showed no effect. **(f) ***EDN3 *mRNA expression analysis as determined by real-time polymerase chain reaction before (-) and after (+) the demethylating treatment illustrates strong re-expression in those cell lines that were substantially demethylated (MCF7: 28-fold; MDA-MB231: 47-fold) as compared with cell lines showing weaker demethylation (BT20: 3-fold; MCF12A: 5-fold) or unmethylated cells (MCF10A: 1.6-fold; SKBR3: 1.0-fold). bp, base pairs; CpG, cytosine-phosphate-guanine dinucleotide; DAC, 5-aza-2'-deoxycytidine; EDN, endothelin; GC, guanine-cytosine content; HMEC, human mammary epithelial cells; PBL, peripheral blood lymphocyte; Plac, placental tissue; TSA, trichostatin A.

Since promoter hypermethylation is responsible for transcriptional silencing of important tumour suppressor genes in various human cancer types [30], we searched all three EDN genes for the presence of CpG islands in their promoter region. A region of high CpG density in the *EDN3 *nucleotide sequence was identified as a CpG island (Figure 4a), whereas the CpG density in *EDN1 *and *EDN2 *is lower and does not define a CpG island under the selected criteria. To analyse the methylation status of the *EDN3 *CpG promoter (Figure 4b), we performed MSP with DNA after bisulphite treatment from non-malignant human tissues and three non-malignant and four malignant breast cell lines. The utilised MSP primers were tested on a dilution series of poly-methylated DNA, which revealed a sensitivity of 0.01 (1%) in detecting methylated *EDN3 *DNA molecules in a background of unmethylated *EDN3 *DNA molecules (Figure 4c). We observed no methylation of the *EDN3 *promoter in HMEC cells, human placental tissue, peripheral blood lymphocytes (Figure 4c) or non-malignant MCF10A cells (Figure 4e). A weak methylation signal was detected in non-malignant MCF12A cells. Of the malignant breast cell lines, MDA-MB231 and MCF7 harboured a methylated *EDN3 *promoter. In BT20 cells, both unmethylated and methylated *EDN3 *promoter sequences could be detected, whereas *EDN3 *was unmethylated in SKBR3 cells. Next, we analysed the association between *EDN3 *expression and promoter methylation in six breast cell lines by *in vitro *demethylating their DNA and assessing *EDN3 *mRNA expression after the treatment. A clear conversion of methylation could be observed in cell lines that were originally methylated in the *EDN3 *promoter region (that is, in MDA-MB231 and MCF7 cells) (Figure 4e), resulting in 47-fold and 28-fold increases, respectively, in *EDN3 *mRNA expression (Figure 4f). In BT20 cells, however, the demethylating effect was weaker and led to a 3-fold induction of *EDN3 *transcription. In weakly methylated MCF12A cells, the conversion of methylated alleles induced *EDN3 *expression by 5-fold, whereas no substantially altered change of *EDN3 *expression could be detected in unmethylated MCF10A (1.6-fold) or SKBR3 (1.0-fold) cells.

### Frequent *EDN3 *promoter methylation in primary breast carcinomas

**Figure 5:**
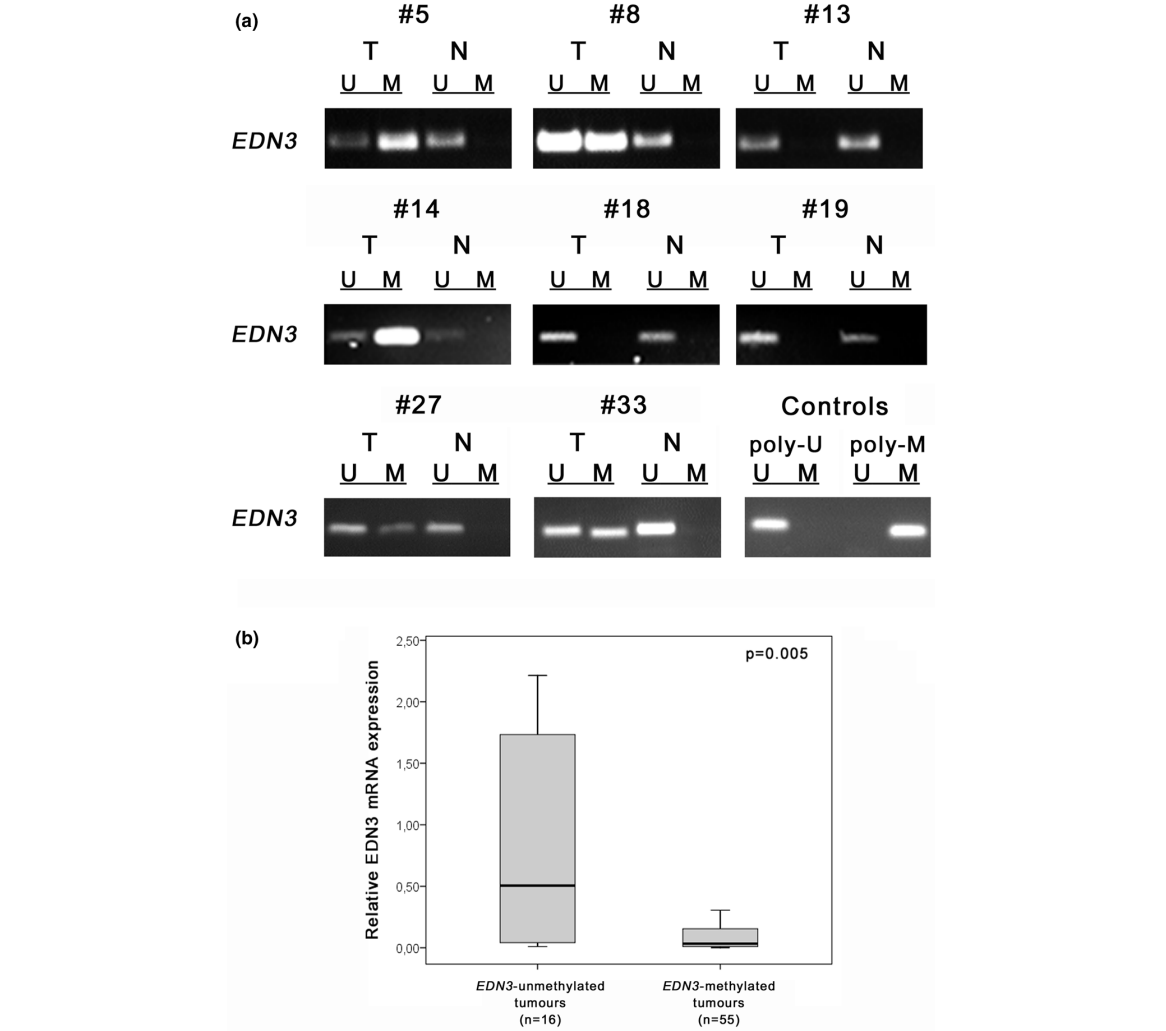
*EDN3 *promoter methylation in primary breast cancer. **(a) **Methylation-specific polymerase chain reaction (MSP) was performed on bisulphite-treated DNA from breast carcinomas (T) and matching normal breast tissue (N). Representative MSP results from eight patients are shown. MSP controls were commercially available poly-unmethylated (poly-U) or *in vitro *poly-methylated (poly-M) DNA. **(b) **Box plot demonstrating the significant association between *EDN3 *promoter methylation and *EDN3 *mRNA expression in these tissues. For 71 samples, *EDN3 *expression has been assessed in parallel to *EDN3 *promoter methylation. *EDN3*-methylated tumours show significant downregulation as compared with *EDN3*-unmethylated tumours (*P *= 0.005, Mann-Whitney *U *test). Horizontal lines indicate group medians, and boxes indicate 25% to 75% quartiles, range, peak and minimum. EDN3, endothelin-3; M, methylated; U, unmethylated.

Next, we analysed *EDN3 *promoter methylation in primary breast cancer as well. In total, 89 of 128 breast carcinoma samples (69.5%) showed *EDN3 *promoter methylation (for example, #5 in Figure 5a). The remaining breast carcinoma samples (39 of 128; 30.5%) were not affected by this epigenetic modification (for example, #13 in Figure 5b). None of the 17 normal breast tissues exhibited *EDN3 *promoter methylation. Cancerous tissues yielded a PCR product with primers specific for the unmethylated *EDN3 *promoter sequence in all cases, due to non-malignant contaminants (stromal cells and endothelial cells) present in the bulk tumour tissue, as has also been described by Suzuki and colleagues [31].

We next aimed to analyse whether *EDN3 *promoter methylation was associated with *EDN3 *mRNA expression in these tissues. For 71 breast cancer specimens, both *EDN3 *methylation and *EDN3 *mRNA expression have been investigated in parallel. Figure 5b shows the distribution of *EDN3 *mRNA expression among these two groups. While carcinoma samples with unmethylated *EDN3 *promoter exhibited similar *EDN3 *mRNA expression as compared with normal breast tissues (Figure 1e), breast carcinomas with *EDN3 *methylation exhibited a significant downregulation of *EDN3 *mRNA expression as compared with *EDN3 *unmethylated samples (*P *= 0.005, Mann-Whitney *U *test).

Finally, we asked whether *EDN3 *promoter methylation may be of clinical relevance in human breast cancer, as we have previously found for EDN3 protein expression. In a univariate analysis, the *EDN3 *methylation status in breast carcinomas was not associated with patient age at diagnosis, tumour size, lymph node metastasis, histological grade, histological type or oestrogen or progesterone receptor positivity (Table 7). In contrast to EDN3 protein expression, *EDN3 *promoter methylation was significantly associated neither with patient CSS (*P *= 0.703) nor with patient DFS (*P *= 0.632) (data not shown).

**Table 7:** Clinicopathological parameters in relation to *EDN3 *promoter methylation

Variables	*EDN3 *promoter			
	Number^a^	Unmethylated (percentage)	Methylated (percentage)	*P *value^b^

Total	128	39 (30.5)	89 (69.5)	
Clinicopathological factors				
Age at diagnosis				
≤ 59 years	64	22 (34)	42 (66)	0.443
> 59 years	64	17 (29)	47 (71)	
Tumour size^c^				
pT1	44	15 (34)	29 (66)	0.680
pT2 to pT4	72	21 (29)	51 (71)	
Lymph node status^c^				
pN0	57	16 (28)	41 (72)	0.676
pN1 to pN3	51	17 (33)	34 (67)	
Histological grade				
G1 and G2	68	18 (26)	50 (74)	0.310
G3	49	18 (37)	31 (63)	
Histological type				
Ductal	103	32 (31)	71 (69)	0.849
Lobular	15	5 (33)	10 (67)	
Other	6	1 (17)	5 (83)	
Immunohistochemistry				
Oestrogen receptor				
Negative (IRS^d ^≤ 2)	33	11 (33)	22 (67)	0.655
Positive (IRS > 2)	85	24 (28)	61 (72)	
Progesterone receptor				
Negative (IRS^d ^≤ 2)	33	11 (33)	22 (67)	0.655
Positive (IRS > 2)	85	24 (28)	61 (72)	

^a^Only female patients with primary invasive breast cancer were included. ^b^Fisher exact test (two-sided). ^c^According to the UICC (Union Internationale contre le Cancer): *TNM Classification of Malignant Tumours *[19]. ^d^Immunoreactivity score (IRS) according to Remmele and Stegner [24]. EDN3, endothelin-3.

## Discussion

The involvement of EDNs in tumourigenesis has been described in several reports [4,10,15]. In contrast to the potentially oncogenic role of EDN1 and EDN2, there is still little knowledge about the role of EDN3 in cancer initiation or progression. A recent study demonstrated abundant expression of EDN1 and EDN2 but complete absence of EDN3 expression in a representative set of human breast cancer cell lines [12]. Because we have previously found that *EDN3 *mRNA expression is downregulated in primary breast carcinomas as compared with normal breast tissues [27,28], we aimed in this report to provide the first comprehensive analysis of EDN3 expression and its potential implication in human breast cancer.

Initially, we screened various non-malignant epithelial tissues for *EDN3 *mRNA expression and also analysed its expression using a breast cancer cDNA dot blot array. Besides abundant expression in several human tissues, *EDN3 *was strongly expressed in normal breast samples, providing evidence for a functional role in epithelial tissues such as the mammary gland. In contrast, most matched breast carcinomas showed diminished EDN3 mRNA expression both on the cDNA dot blot array and by real-time PCR analysis. This finding supports the current evidence that EDN3 may exert a functional role divergent to that of EDN1/EDN2 in the human mammary gland [18]. A further TMA analysis revealed that EDN3 protein is abundantly expressed in normal breast whereas its expression is reduced in a large fraction of breast carcinomas. Frequency differences may arise due to the use of different techniques (real-time PCR versus IHC) on separate tumour cohorts (fresh frozen versus FFPE) and accomplishing different scoring systems. Loss of EDN3 protein expression was not associated with relevant clinicopathological factors. For instance, it occurred with almost equal frequency among all tumour sizes (pT1 to pT4), suggesting that it may be an early event in the development of infiltrating breast carcinoma. Since EDN3 is thought to counterbalance the effects mediated by EDN1 and EDN2 [4,18], we propose that loss of EDN3 expression could actively enhance overexpression of the ET-axis. Recently, upregulation of ET-axis members was found to be associated with higher histological grade, lymph node metastasis and lymphovascular invasion in breast cancer [5] and also with advanced tumour progression in ovarian cancer [32], prostate cancer [33], Ewing sarcoma and neuroblastoma [11]. A systematic expression analysis on larger breast carcinoma cohorts and metastatic deposits is now required, including all three EDNs and EDNRA/EDNRB. This will unravel in detail the inter-relationship between EDN3 expression loss and upregulation of EDN1/2 and EDNRA/B as well as its association with breast tumour progression. In our study, loss of EDN3 expression was associated with adverse patient outcome. So far, overexpressions of EDN1 and EDNRA were already reported as being associated with impaired survival in breast cancer [5,34]. Our findings support the view that an imbalanced ET-axis is of pivotal relevance in breast cancer biology and that EDN3, unlike other members of the ET-axis, may represent a novel tumour suppressor gene in the human mammary gland.

Addressing the molecular cause by which EDN3 expression becomes abrogated, we found that the *EDN3 *gene promoter, unlike *EDN1 *and *EDN2*, contains a CpG island as a potential substrate to aberrant hypermethylation and consequently gene inactivation. Indeed, we detected *EDN3 *promoter methylation in cancerous breast cell lines in functional association with loss of *EDN3 *mRNA expression. Moreover, a hypermethylated *EDN3 *promoter was also detected in 70% of breast carcinoma specimens in significant association with loss of *EDN3 *expression. We therefore conclude that aberrant *EDN3 *methylation is a tumour-specific event and the predominant mechanism leading to EDN3 expression loss in breast cancer. However, it remains elusive why patient survival was not associated with *EDN3 *methylation as it was with loss of EDN3 protein expression. In fact, only very few studies detected such outcome association on both molecular levels (for example, for SFRP1 [22,35] or ITIH5 [36]), probably due to considerable sensitivity differences of the available detection techniques as well as further genetic or epigenetic alterations contributing to the loss of a gene's expression. Interestingly, *EDNRB *was previously described to be methylated in numerous tumour entities, such as lung, colon, prostate, bladder, kidney, liver, oesophageal, nasopharyngeal cancer and leukemia [37-45], but to the authors' knowledge, never in gynaecological tumours. So far, there has been no evidence that *EDNRB *becomes methylated in breast carcinomas since a previous study demonstrated strong EDNRB expression in all invasive ductal carcinoma samples and in all analysed cancerous breast cell lines [4]. Notably, an ET-axis expression pattern similar to that of breast cancer was recently found in cervical cancer; that is, upregulation of EDN1, EDN2, EDNRA and EDNRB expression was accompanied by downregulation of EDN3 expression in cancerous cervix as compared with normal cervical epithelium [46]. This suggests that a decrease of EDN3 expression accompanied by an increase of EDNRB expression may be a particular feature of gynaecological tumour entities.

Since the ET-axis represents crucial decisive elements for the direction of tumour growth, invasion and neo-angiogenesis, it provides a promising intervention point for molecular targeted therapies. EDNR antagonists have been proven as potent and specific ET-axis inhibitors that block cellular pathways implicated in tumour growth. The drug bosentan, which targets both EDNRA and EDNRB, inhibits tumour growth, vascularisation and bone metastasis in breast cancer [47]. Atrasenatan, targeting EDNRA, is capable of inhibiting proliferation and cancer growth-promoting processes [48,49]. In addition, blockers of EDNRA resensitised cancer cells to paclitaxel-induced apoptosis, as observed in ovarian, prostatic, cervical and nasopharyngeal cancer cell lines [49-51] as well as in primary ovarian and breast cancer [5,52]. Our study adds a novel aspect to therapeutically targeting the ET-axis in breast cancer. Since the epigenetic lock of the *EDN3 *gene is potentially reversible by DNA methyltransferase (DNMT) or histone acetyltransferase (HDAC) inhibitors or both, these drug classes may provide a future option in a combined treatment consisting of a decrease in EDN1/2 signalling by blocking EDNRs together with the reactivation of EDN3 expression by DNMT and HDAC inhibitors. Apparently, molecular rebalancing of the ET-axis in cancerous cells may be most efficiently achieved by targeting all deregulated axis molecules.

## Conclusions

In summary, our study contributes to the understanding of deregulated EDN signalling commonly observed in human tumours. EDN3 expression, in contrast to abundant EDN1 and EDN2 expression, becomes frequently inactivated by promoter methylation in human breast cancer, potentially causing aberrant activation of the ET-axis, which in turn may promote this disease. We therefore conclude that *EDN3 *may be an interesting future target for an epigenetic therapy. Forced EDN3 re-expression by DNA demethylation agents in conjunction with inhibitors of EDNRs may rebalance ET-axis-mediated cellular signalling to a more normal status, thus having a therapeutic impact in human breast cancer.

## Abbreviations

bp: base pair(s); CI: confidence interval; CPA: cancer profiling array; CpG: cytosine-phosphate-guanine dinucleotide; CSS: breast cancer-specific survival; C_T_: cycle threshold; DAC: 5-aza-2'-deoxycytidine; DFS: disease-free survival; DNMT: DNA methyltransferase; EDN: endothelin; EDNR: endothelin receptor; ET-axis: endothelin axis; FC2: fold change of greater than 2; FFPE: formalin-fixed paraffin-embedded; GAPDH: glyceraldehyde-3-phosphate-dehydrogenase; HDAC: histone acetyltransferase; IHC: immunohistochemistry; IRS: immunoreactivity score; MSP: methylation-specific polymerase chain reaction; MTN: multiple-tissue Northern (blot); PCR: polymerase chain reaction; SD: standard deviation; TMA: tissue microarray; TSA: trichostatin A.

## Competing interests

The authors declare that they have no competing interests.

## Authors' contributions

FW participated in the conception and design of the study and carried out the gene expression analyses, immunohistochemical studies and methylation experiments. JV performed statistical evaluations, participated in data interpretation and wrote the manuscript. OG provided expertise in DNA methylation analysis and critically revised the manuscript. AH provided clinical samples and clinicopathological data, performed data interpretation and critically revised the manuscript. ME participated in data interpretation and critically revised the manuscript. RK participated in the design and coordination of the study and critically revised the manuscript. ED planned and coordinated the study and critically revised the manuscript. All authors read and approved the final manuscript.

## Supplementary Material

Additional data file 1 A PDF file that demonstrates the specificity of the applied EDN3 antibody by use of competitive blocking peptide in immunohistochemistry. Click here for file
