# Supplementary material for: Frequent loss of endothelin-3 (EDN3) expression due to epigenetic inactivation in human breast cancer
Source: Breast Cancer Res. 2009 Jun 15;11(3):R34. doi: 10.1186/bcr2319 (PMC2716502; doi:10.1186/bcr2319)
Supplement: Additional data file 1 — A PDF file that demonstrates the specificity of the applied EDN3 antibody by use of competitive blocking peptide in immunohistochemistry. [file bcr2319-S1.pdf]

(a)

(b)

(c)

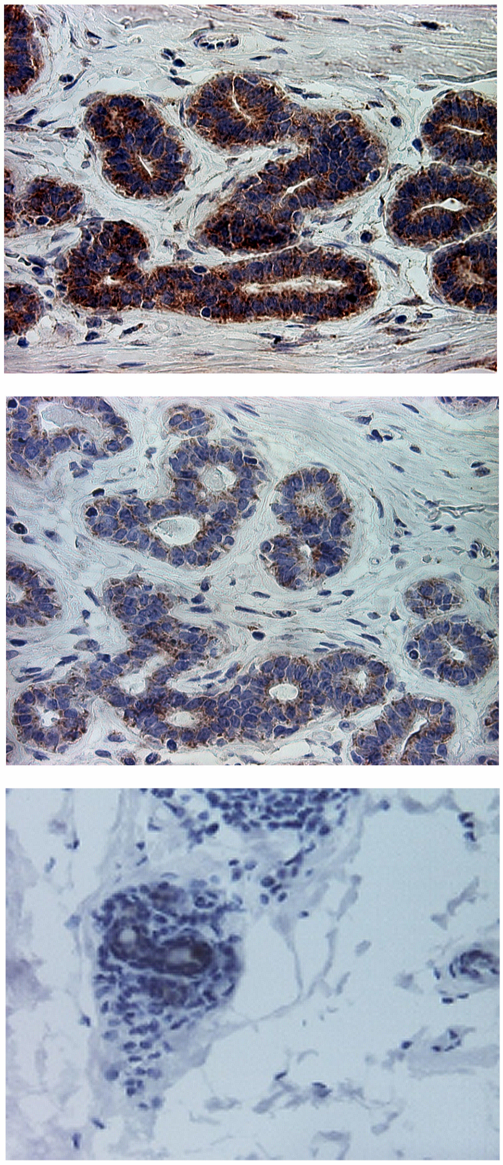

Additional data file 1: Specificity of the applied EDN3 antibody. (**a**) Normal breast tissue stained with the antibody as indicated in the Materials and Methods section. (**b**) The prior incubation of the antibody with the respective blocking peptide (Santa Cruz Biotechnology Inc., sc-21628 P) for 2 hours strongly reduces staining intensity. (**c**) Normal breast tissue as negative control, in which the primary antibody has been omitted. Magnifications: 200x.
